# Retraction

**DOI:** 10.29252/ibj.24.6.404

**Published:** 2020-11

Due to the redundant information presented in "MS14 Down-Regulates Lipocalin2 Expression in Spinal Cord Tissue in an Animal Model of Multiple Sclerosis in Female C57BL/6. Iran Biomed J. 2014 October; 18(4): 245-249; DOI: 10.6091/ibj.1375.2014" and the paper published in the Iran Red Crescent Journal: "MS14, a Marine Herbal Medicine, an Immunosuppressive Drug in Experimental Autoimmune Encephalomyelitis. Iran Red Crescent Med J. 2014 July; 16(7): e16956", the editor/publisher primarily declared a notice of "partial retraction" (dated 26 August, 2020). But since the amount of redundant information was later found very significant (Figure-1 and the graph in Figure-2) and its omission would not leave a credible paper, IBJ was obliged to issue a notice of full retraction on 1 November 2020. The authors are not in agreement with this decision.

